# Mediating effects of depression on sleep disturbance and frailty in older adult type 2 diabetes patients in the community

**DOI:** 10.3389/fpubh.2023.1237470

**Published:** 2023-11-28

**Authors:** Xushu Chen, Mengdan Liu, Qin Ma, Xin Liu, Xueping Peng, Changjiu He

**Affiliations:** ^1^School of Nursing, Chengdu Medical College, Chengdu, China; ^2^The Clinical Hospital of Chengdu Brain Science Institute, MOE Key Laboratory for Neuroinformation, University of Electronic Science and Technology of China, Chengdu, China

**Keywords:** frailty, sleep disturbance, depression, type 2 diabetes, older adult

## Abstract

**Introduction:**

With the progressive aging of the population, frailty is now a significant challenge in geriatrics research. A growing amount of evidence suggests that sleep disturbance and depression have independent effects on frailty, although the underlying mechanisms are not yet clear. This study aimed to investigate the mediating role of depression in the relationship between sleep disturbance and frailty in older adult patients with type 2 diabetes (T2DM) in the community.

**Method:**

Purposive sampling was used to collect face-to-face data from 342 community-dwelling T2DM patients in Chengdu, Sichuan Province, China, between February and May 2023. The Pittsburgh Sleep Quality Index (PSQI) scale was used to evaluate sleep quality, the Simple Geriatric Depression Scale (GDS-15) was used to evaluate depressive symptoms, and the FRAIL Scale (FRAIL) was used to evaluate frailty. Linear regression equation and bootstrap self-sampling were used to verify the mediating role of depressive symptoms in sleep disturbance and frailty.

**Result:**

The study found that sleep disturbance had a direct positive effect with frailty [β = 0.040, 95% CI: (0.013, 0.069)]. Additionally, depression had a direct positive effect on frailty [β = 0.130, 95% CI: (0.087, 0.173)], and depression was found to partially mediate the relationship between sleep disturbance and frailty.

**Conclusion:**

Poor sleep quality and frailty are common in patients with T2DM. To reduce the frailty of older adult T2DM patients, all levels of society (government, medical institutions, and communities) must pay more attention to mental health. A variety of interventions should be considered to improve sleep quality and depression, which in turn may prevent or control frailty.

## Introduction

The aging population presents a significant challenge for contemporary societies and has garnered greater global attention. It is projected that by 2050, a fifth of the population will be over the age of 65 (26.1%) (1, 2). China has the largest population of older adult citizens and is now one of the fastest aging nations globally (3). The rise in the aging population has coincided with the growing prevalence of several chronic ailments, especially diabetes (4). With the findings of the survey conducted by the International Diabetes Federation (IDF) (5), the current global population of adults with diabetes is ~537 million. China has been identified as the country with the largest number, with over 110 million diabetic patients (6). Type 2 diabetes mellitus (T2DM) accounts for more than 90% (7). The prevalence of T2DM is 23.9% in people aged 60–69 and 27.3% in those aged 70 and over (8). It can be seen that the situation regarding the prevalence of diabetes in the country has become even more critical.

Frailty poses a significant challenge to older adults as they age (9). Frailty is a complex concept that must account for the intricate interplay of various factors often manifesting in three dimensions: physical, psychological, and social frailty (10). In China, the older adult patient population with T2DM had 22.7% higher frailty detection rates than patients without T2DM (11, 12). Moreover, frailty may heighten the risk of falls, disability, and mortality in older adult patients with T2DM, jeopardizing the physical and mental health of older adults and causing a significant economic strain on families, society, and healthcare (13). These findings highlight the significance of prioritizing frailty management in older adult T2DM patients amidst a rapidly aging population.

Previous studies showed that sleep disturbance may associated with frailty (14), But the underlying mechanism has not yet been revealed. Sleep disturbance impacts many older people with T2DM (15). Older adult people with T2DM have a higher incidence of sleep disturbance due to obstructive sleep apnoea or nocturnal symptoms such as hypoglycemia, nocturia, or neuralgia (16, 17). Poor sleep quality can lead to impaired protein synthesis and muscle loss, and these changes are a significant cause of frailty in older people (18, 19). A previous prospective cohort study found a high risk of frailty in older adults with sleep problems (20). Older adults with sleep problems were 49.95 times more likely to be frail than those without sleep problems (21). Several studies have shown a positive association between sleep problems and frailty (21, 22). Therefore, sleep disturbance may be one of the factors affecting frailty among T2DM patients. In this study, we focused on sleep disturbance in T2DM patients and hypothesized that their sleep disturbance would be positively correlated with their frailty. In addition, sleep disturbances may associated with depression and can lead to low mood (23).

Depression is a common and complex mental illness. Globally, depression is one of the most common mental illnesses in people 65 years and older, affecting one in seven older adults (24). Studies have shown that sleep disturbance is a risk factor for depression in older patients with T2DM (25). Previous study of older adult people with T2DM, sleep disturbance was positively associated with depression. There is also evidence that sleep disturbance is strongly associated with depression, with an increasing prevalence of sleep disturbance and higher detection rates of depression in older adults (26). Additionally, Almeida et al. demonstrated that depression may be associated with frailty among older adult T2DM patients (27).

People with depression are more likely to be frailty (28, 29). A previous study found that older adult, depression people with T2DM are at high risk of frailty (30). Depression increases the risk of adverse outcomes with older adult T2DM patients in frailty. Compared with non-depressed people with diabetes, people with depression have a significantly lower quality of life in physical and mental functioning, which can lead to disability and death (31). As a result, we further predicted depression as a mediator in the relationship between sleep disturbance and frailty among community-dwelling older adult T2DM patients.

A review of previous studies found that most previous studies in this area have examined the relationship between sleep disturbance, depression, and frailty. However, the combined effects of these factors on frailty and the underlying mechanisms of these relationships were unclear. In addition, few studies have examined frailty in a specific group—older adult T2DM patients. Therefore, this study investigated the association between sleep disturbance, depression, and frailty in community older adult T2DM patients in Chengdu and the possible underlying mechanisms.

Based on previous studies, we propose 4 hypotheses: (1) Sleep disturbance has a direct positive effect on frailty; (2) Sleep disturbance has a direct positive effect on depression; (3) Depression has a direct positive effect on frailty; (4) The relationship between sleep disturbance and frailty is mediated by depression. Our findings identified the critical factors for preventing and controlling frailty in older adult T2DM patients in the community, which is essential for reducing frailty and improving the health of T2DM patients.

## Materials and methods

### Participants

This was a cross-sectional study conducted between February 2023 and May 2023. Approval was obtained from the Ethics Committee of Chengdu Fourth Hospital was obtained prior to this study. Our study used purposive sampling method to select two community health centers in Chengdu city, and purposive sampling was applied in the community to select respondents who met the study criteria. Respondents were asked to meet the following inclusion criteria: (1) Diagnosed by the attending physician meeting the diagnostic criteria for T2DM by the World Health Organization (WHO) in 1999 (32); (2) age ≥60 years; (3) diagnosis ≥3 months; (4) no significant (or corrected) impairment of vision, hearing or communication; and (5) voluntary participation and signing an informed consent form. Exclusion criteria were as follows: (1) diagnosed psychiatric disease; (2) presence of severe organic disease intolerant of the survey; (3) patients with severe acute complications of diabetes mellitus in combination were excluded. In the end, a total of 342 questionnaires were distributed and, after excluding invalid questionnaires, 319 valid questionnaires were finally obtained and 93% (319/342) valid responses were obtained and analyzed. In addition, we refer to the sample size estimation formula for cross-sectional studies (33): $\text{n}=\frac{Z^{2}P\left(1-P\right)}{d^{2}}$. Where n is the sample size, *Z* is the statistic corresponding to the confidence level, *P* is the expected prevalence, and *d* is the precision. We assumed a confidence level of 95.0%, an expected prevalence of frailty of 19.2% based on the prevalence of frailty of T2DM in the community of Xianning City, Hubei Province, China, as reported by Linglin Kong et al. and a precision of 5.0%. Therefore, taking into account the formula, we obtained *n* ≈ 239. Taking into account a questionnaire loss rate of 20%, the sample size was calculated to be at least 287 cases.

### Measures

All measures in this study would be conducted in Chinese.

### Basic sociodemographic data

A self-designed questionnaire was adopted, including gender, age, ebody fat index (BMI), education, marital status, residential status, monthly income., physical activity, disease duration, and complications. BMI is calculated by dividing weight (kg) by the square of height (m), with a value of 18.5–23.9 as normal and a value of < 18.5 or >23.9 as abnormal BMI (34). Marital status is classified as married and single. Being single includes being unmarried, widowed, and divorced.

Physical Activity is exercising more than 3 times or 300 min a week (35). Types of exercise include: walking, jogging/cycling, Tai chi, swimming, apparatus sports, dance exercises (square dancing, aerobics, yoga), and ball games (36). Complications include peripheral neuropathy, retinopathy, diabetic nephropathy, diabetic foot, cerebrovascular, and cardiovascular disease (37).

### Frailty

Frailty was assessed using the frailty scale (38). The scale consists of 5 questions: (1) Fatigue: Have you felt tired frequently in the past 4 weeks? (2) Low resistance: Is it difficult to climb stairs without AIDS and help from others? (3) Low walking amount: Is it difficult to walk a block of about 500 m without AIDS and help from others? (4) Weight loss: Have you lost at least 5% of your body weight in the past year? (5) Diseases: Have you had five or more diseases? The scale is scored on a scale of 0–5. If the old person answered “yes”, they got a “1” and if they answered “no”, they got a “0”. Scores of 0, 1–2, and 3–5 represent robust, prefrail, and frail, respectively. Dong et al. (39) applied its translation to the older adult in Chinese communities and found that the frailty scale had good validity and reliability (40). It showed good validity and test–retest reliability in Chinese patients with type 2 diabetes (41).

### Sleep disturbance

Sleep quality in patients with T2DM was assessed using the Pittsburgh Sleep Quality Index (PSQI) (42). The scale was compiled by Dr. Buysse in 1989. The PSQI scale included sleep quality, sleep time, sleep duration, sleep efficiency, sleep disorders, hypnotic drugs, and daytime dysfunction. Nineteen self-rated items were scored and composed of 7 components. Each component was scored on a scale of 0 3. The cumulative score of each component was the total PSQI score, with the total score ranging from 0 to 21, and PSQI score > 7 was considered as sleep disorder. Cronbach's α coefficient in this study was 0.79. The Chinese version of PSQI has been proven to be reliable and valid in the community-dwelling older population (43).

### Depression

The Geriatric Depression Scale-15 (GDS-15) was developed from the Geriatric Depression Scale developed by Reisberg et al. in 1982 (44). GDS-15 consists of 15 items, each with “yes” and “no” options. The total score is 0–15 points, among which items 1, 5, 7, and 11 are scored in reverse. A score of 0–4 indicates no symptoms of depression, and a score of ≥5 indicates symptoms of depression. The Chinese version of GDS-15 is a reliable and valid screening tool for assessing geriatric depressive symptoms in the Chinese population (45). The Cronbach α coefficient of this scale in this study was 0.77.

### Procedure

To ensure consistency in survey methodology, all information was collected in face-to-face interviews by uniformly trained investigators with a medical background. All items were explained in an unbiased manner by the investigator and, after written informed consent was obtained, all participants were assured that their responses would be anonymous and confidential. The questionnaire also explained the purpose of the survey, how to complete it, and the promise of confidentiality. Finally, face-to-face data collection took place and questionnaires were returned on the spot.

### Statistical analysis

The data was entered using Epidata software. Data quality was ensured by consistent coding of the questionnaires, and separate entry by two individuals. All analyses were conducted using IBM SPSS Statistics version 25.0 (Armonk, NY, United States). The significance level for the two-tailed test to α = 0.05. We expressed continuous data as mean ± standard deviation and categorical data as (*n*) and percentage (%). We tested the correlation between variables using Pearson correlation analysis. In a similar way, McKinnon's four-step approach (46) was used in our study to analyze the mediating role with four specific criteria that had to be met: (1) There was a significant association between the independent variable (sleep disturbance) and the dependent variable (frailty); (2) There was also a significant association between the independent variable (sleep disturbance) and the mediator variable (depression); (3) There was a significant correlation between the mediator variable (depression) and the dependent variable (frailty) after adjusting for the control of the independent variable (sleep disturbance); (4) The coefficient of indirect correlation between the independent variable (sleep disturbance) and the dependent variable (frailty) through the mediator variable (depression) is significant. The first three steps were individually tested by means of a linear regression equation with α_in_ = 0.05 and α_out_ = 0.01. Finally, the mediation effect was analyzed using the PROCESS version 3.0 macro for SPSS (model 4), with bootstrap 5,000 self-sampling to verify the final condition. Statistical significance is indicated when 0 is excluded from the 95% confidence interval.

## Results

### Common method biases test

We used Harman's Single Factor Test to examine the common method bias in the study. The analysis showed that the first common factor analyzed explained only 24.45% (< 40%) of the variance. This suggests that there was no significant common method bias in the study, even though we used the questionnaire.

Table 1 shows the sociodemographic characteristics of the older adult T2DM patients. Of the 319 eligible patients, 166 (52.0%) were female and 153 (48.0%) were male. The mean age of the participants was (72.54 ± 6.19) years. One hundred and thirty-four (42.0%) of the participants had a normal BMI and 185 (58.0%) had a high or low BMI. The educational background of the participants was as follows: 134 (42.0%) had a junior high school education or less, and 185 (58.0%) had a high school education or more. The participants' marital status was as follows: 67 (21.0%) were single and 252 (79.0%) were married. Of the total number of patients surveyed, 25 (7.8%) were living alone and the remaining 194 (30.7%) were not living alone. There were 134 patients (42.0%) with a monthly personal income of < $2,000 and 185 patients (58.0%) with a monthly personal income of more than $2,000. There were 257 (80.6%) participants who exercised regularly, while 62 (19.4%) exercised less. There were 171 (53.6%) patients with a disease duration of < 10 years and 148 (46.4%) participants with a disease duration of >10 years. Finally, 127 (39.8%) patients had chronic complications of diabetes and the remaining 192 (60.2%) did not.

**Table 1:** Social demographic features and differences of T2DM patients frailty scores.

**Variables**	**Mean ±SD (range) *N* (%)**
**Gender**	
Male	153 (48.0)
Female	166 (52.0)
Age	72.54 ± 6.19
**BMI (kg/m** ^2^ **)**	
18.5–23.9	134 (42.0)
< 18.5 or >23.9	185 (58.0)
**Education level**	
Primary school or below	134 (42.0)
Junior school and above	185 (58.0)
**Marital status**	
Single	67 (21.0)
Married	252 (79.0)
**Living state**	
Live alone	25 (7.8)
Live with others	294 (92.2)
**Monthly personal income (CNY)**	
< 2,000	134 (42.0)
≥2,000	185 (58.0)
**Exercise**	
No	62 (19.4)
Yes	257 (80.6)
**Duration of diabetes**	
< 10 years	171 (53.6)
≥10 years	148 (46.4)
**Diabetic chronic complications**	
No	192 (60.2)
Yes	127 (39.8)

### Correlations of the study variable

Table 2 shows Spearman's correlations for the study variables. Sleep disturbance (*r* = 0.319, *p* < 0.01) and depression (*r* = 0.441, *p* < 0.01) were significantly positively correlated with frailty. In addition, sleep disturbance was significantly positively correlated with depression (*r* = 0.378, *p* < 0.01).

**Table 2:** The statistical descriptions and associations among study variables.

**Variables**	**Mean**	***SD***	**Sleep disturbance**	**Depression**	**Frailty**
Sleep disturbance	7.81	4.42	-		
Depression	2.86	2.77	0.378^**^	-	
Frailty	0.85	1.11	0.319^**^	0.441^**^	-

^**^P < 0.01.

### The mediation effect analysis

**Figure 1:**
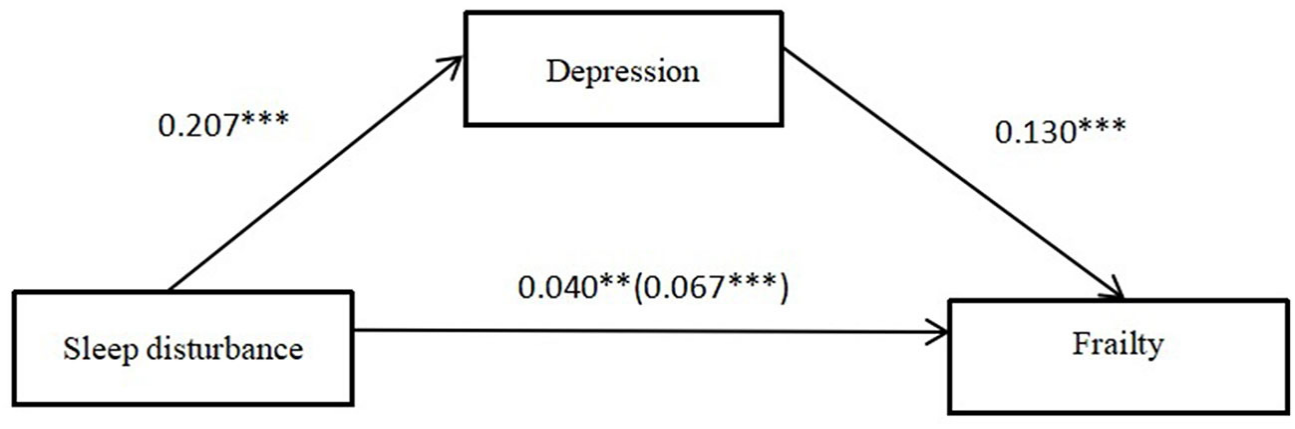
Model diagram of the effect of sleep disturbance on frailty (**p < 0.01, ***p < 0.001).

Table 3 reports the analysis of mediating effects among the variables. After controlling for variables, there was a significant direct effect of sleep disturbances on frailty [β = 0.067, CI (0.041, 0.094)]. In addition, sleep disturbance had a significant positive effect on depression [β = 0.207, CI (0.141, 0.273)]. Depression were significantly effected frailty [β = 0.130, CI (0.087, 0.173)]. Furthermore, the effect of sleep disturbance on frailty was statistically significant even when the mediating variable was included (see Table 4; β = 0.040, CI (0.014, 0.067)]. Based on the bootstrap 95% CI (see Table 4; depression = 0.033, 95% CI = 0.019 ~ 0.050) not containing 0, it can be concluded that depression partially mediates the relationship between sleep disturbance and frailty. Thus, hypothesis was confirmed. Note that the final mediation model is shown in Figure 1.

**Table 3:** Regression analysis among study measures.

**Variables**	**β**	***t***	***P***	**LLCI**	**ULCI**	***R* ^2^**	***F***
**Result variable: depression**							
Predictor sleep disturbance	0.207	6.199	< 0.001	0.141	0.273	0.181	7.405
**Result variable: frailty**							
Predictor sleep disturbance	0.040	2.981	0.003	0.014	0.067	0.259	10.259
Mediator depression	0.130	5.967	< 0.001	0.087	0.173		
**Result variable: frailty**							
Independent variable sleep disturbance	0.067	4.999	< 0.001	0.041	0.094	0.175	7.150

Controlling for gender, age, BMI, education, marital status, living state, monthly income, exercise, duration of diabetes, diabetic chronic complications.

**Table 4:** Bootstrap analysis of the chain mediating model.

**Path**	**Effect**	**Boot SE**	**Boot**	**Boot**	**Effect ratio**
			**LLCI**	**ULCI**	
Total effect	0.075^***^	0.014	0.040	0.094	100%
Direct effect	0.040^**^	0.014	0.013	0.069	59.70%
Indirect effect	0.033^***^	0.007	0.019	0.050	40.30%

Controlling for gender, age, BMI, education, marital status, living state, monthly income, exercise, duration of diabetes, diabetic chronic complications.

^***^P < 0.001.

^**^P < 0.01.

## Discussion

This study aimed to examine the relationship between sleep disturbance, depression, and frailty in older adult T2DM patients among the community in Chengdu, China. To the best of our knowledge, this is the first study to report the mediating role of depression between sleep disturbance and frailty in older adult T2DM patients in the community. The results of this study may provide possible informations and directions for the formulation and implementation of intervention strategies and measures to improve frailty in older adult T2DM patients.

As speculated, our results suggest that sleep disturbance positively effects the frailty of older adult T2DM patients. This is consistent with existing studies (47). One possible explanation is that chronic sleep disturbance in older adult T2DM patients may lead to multisystem disease and dysfunction. Sleep disturbance often lead to daytime sleepiness and increased fatigue in older patients (14). It may also reduce social interaction or lead to a lack of social interaction in older adult T2DM patients. Over time, older adult T2DM patients may experience a significant decline in muscle strength and fitness and a lack of sensory stimulation, eventually leading to frailty (48). Therefore, providing adequate sleep security is an effective strategy to reduce frailty in older adult T2DM patients. Furthermore, lifestyle modifications, appropriate diabetes management, and increased social interaction may help to reduce frailty among older adult T2DM patients with poor sleep quality (49, 50).

After controlling for variables, depression plays a mediating role between sleep disturbance and frailty, as supported by a previous study (51). Depression is a crucial mechanism by which sleep disturbances effect frailty in T2DM, and sleep disturbances may further lead to frailty by depression in T2DM patients. Hypothesis 4 is supported. Numerous studies have demonstrated a complex biological connection between the duration of sleep and the symptoms of depression (52, 53). Sleep disturbance can cause significant variations in blood glucose levels, leading to challenges in its management (54). It also reduces insulin sensitivity, exacerbating the condition. Moreover, patients often face difficulty managing their diet in the presence of frequent fluctuations in blood glucose levels, leading to helplessness, reduced confidence in the battle against the illness, and depression (55). Older adult T2DM patients with depression have significantly elevated levels of C-reactive protein, interleukin-6 and tumor cytokines, which also contribute to frailty (56, 57). Meanwhile, older adult T2DM patients with depression are significantly less sociable and active and are more likely to have a sedentary lifestyle (58), which further contributes to muscle wasting. And muscle wasting and reduced muscle strength are important markers of frailty (59). In addition, co-morbidities with other chronic conditions can further exacerbate frailty (60). As a result, it is important to address the sleep problems of patients with type 2 diabetes mellitus to prevent the harmful impact of prolonged lack of sleep on their mental health and frailty.

## Conclusion

The study suggests a significant and positive association between sleep disturbance and frailty in older adult T2DM patients living in the Chengdu community, with depression as a mediator. Thus, this study may help to elucidate the pathogenesis of frailty. A high-quality sleep may decrease the risk of depression and, consequently, frailty. Therefore, we suggest the following recommendations. To reduce frailty in T2DM patients, firstly, Government and community should provide more social opportunities for patients and actively encourage their participation in activities. Secondly, nursing managers should provide emotional regulation and management training to older adult T2DM patients in the community, which will improve their ability to cope with depression and increase their resilience. Also, patients with T2DM who need more social support from their families to improve the quality of sleep.

## Limitations

This study has the following limitations: First, the sample size of this study was small. Secondly, the variables of “sleep disturbance,” “depression,” and “frailty” in this study were all subjectively reported by the subjects themselves, which may have information bias. Third, we tested only one mediating variable. Future studies need to further explore the influence of other potential variables, such as social support and self-efficacy, that are associated with frailty. Finally, the sample of this study comes from only two communities in Chengdu. Due to the differences in social culture, economy, and lifestyle, attention should be paid to the universality of the findings.

## Data availability statement

The original contributions presented in the study are included in the article/supplementary material, further inquiries can be directed to the corresponding author.

## Ethics statement

Written informed consent was obtained from the individual(s) for the publication of any potentially identifiable images or data included in this article.

## Author contributions

XC and XP was involved in all aspects of the study and preparation of the manuscript. CH, QM, ML, and XL was involved with the design of the study and preparation of the manuscript. All authors contributed to the article and approved the submitted version.
